# Inter-hospital transfer in patients with acute myocardial infarction in China: Findings from the improving care for cardiovascular disease in China-acute coronary syndrome project

**DOI:** 10.3389/fcvm.2022.1064690

**Published:** 2022-12-08

**Authors:** Danqing Hu, Yongchen Hao, Jun Liu, Na Yang, Yiqian Yang, Zhaoqing Sun, Dong Zhao, Jing Liu

**Affiliations:** Department of Epidemiology, Beijing Anzhen Hospital, Beijing Institute of Heart, Lung and Blood Vessel Diseases, Capital Medical University, Beijing, China

**Keywords:** acute myocardial infarction, inter-hospital transfer, direct admission, early revascularization, in-hospital outcomes

## Abstract

**Background:**

Little is known about the current scenario of inter-hospital transfer for patients with acute myocardial infarction (AMI) in China.

**Methods:**

From November 2014 to December 2019, 94,623 AMI patients were enrolled from 241 hospitals in 30 provinces in China. We analyzed the pattern of inter-hospital transfer, and compared in-hospital treatments and outcomes between transferred patients and directly admitted patients.

**Results:**

Of these patients, 40,970 (43.3%) were transferred from hospitals that did not provide percutaneous coronary intervention (PCI). The proportion of patients who were transferred from non-PCI hospital was 46.3% and 11.9% (*P* < 0.001) in tertiary hospitals and secondary hospitals, respectively; 56.2% and 37.3% (*P* < 0.001) in hospitals locating in low-economic regions and affluent areas, respectively. Compared with directly admitted patients, transferred patients had lower rates of reperfusion for STEMI (57.8% vs. 65.2%, *P* < 0.001) and timely PCI for NSTEMI (34.7%vs. 41.1%, *P* < 0.001). The delay for STEMI patients were long, with 6.5h vs. 4.5h from symptom onset to PCI for transferred and directly admitted patients, respectively. The median time-point was 9 days for in-hospital outcomes. Compared with direct admission, the hazard ratios and 95% confidence intervals associated with inter-hospital transfer were 0.87 (0.75–1.01) and 0.87 (0.73–1.03) for major adverse cardiovascular events and total mortality, respectively, in inverse probability of treatment weighting models in patients with STEMI, and 1.02 (0.71–1.48) and 0.98 (0.70–1.35), respectively, in patients with NSTEMI.

**Conclusion:**

More than 40% of the hospitalized AMI patients were transferred from non-PCI-capable hospitals in China. Further strategies are needed to enhance the capability of revascularization and reduce the inequality in management of AMI.

## Introduction

Acute myocardial infarction (AMI), including ST-segment elevation myocardial infarction (STEMI) and non-ST-segment elevation myocardial infarction (NSTEMI), is the most serious clinical presentation of ischemic heart disease (1). Primary percutaneous coronary intervention (PCI) for patients with STEMI and timely PCI for high-risk or very high-risk patients with NSTEMI are the key strategies for early management recommended by all relevant clinical guidelines (2–4). Unfortunately, many AMI patients initially arrive at hospitals that have no capacity to provide acute treatment, particularly early revascularization. These patients should be transferred to PCI-capable hospitals for further treatment. For AMI patients arriving at non-PCI-capable hospitals, guidelines recommend an inter-hospital transfer to a PCI hospital within 120 min for patients with STEMI and very high-risk NSTEMI, and a same-day transfer for high-risk NSTEMI patients (2–4). In the real world, however, many patients may not be transferred to PCI-capable hospitals within the recommended time.

Some previous studies have reported that a significant proportion of AMI patients experienced inter-hospital transfer (5–8). However, few studies have explored the quality of care among these patients in China, especially for patients with longer treatment delay. Systematic understanding of the characteristics of transferred AMI patients and the impact of inter-hospital transfer on in-hospital case management and outcomes if any, is essential to improve the prognosis of these patients.

We therefore explore the proportion of patients admitted with AMI who were transferred between hospitals, as part of the Improving Care for Cardiovascular Disease in China-Acute Coronary Syndrome (CCC-ACS) project. We also aimed to investigate the characteristics, treatment delay, early revascularization and outcomes of patients undergoing inter-hospital transfer, compared with patients with STEMI and NSTEMI who were admitted directly to a PCI-capable hospital in China.

## Materials and methods

### Study design and population

**FIGURE 1:**
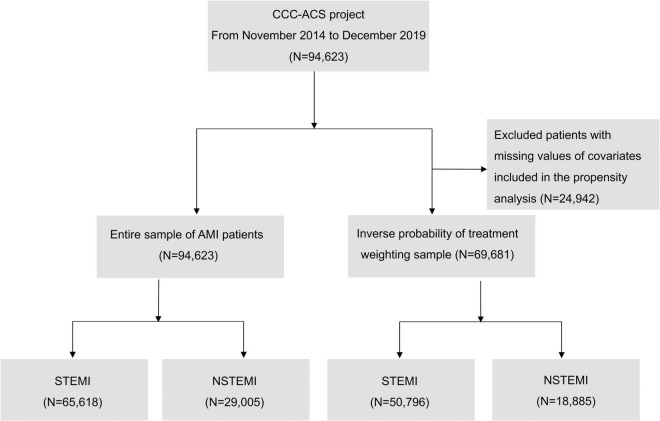
The flowchart of participant inclusion and exclusion. AMI, acute myocardial infarction; NSTEMI, non-ST-segment elevation myocardial infarction; and STEMI, ST-segment elevation myocardial infarction.

The CCC-ACS project is a nationwide quality improvement registry program launched in 2014 as a collaborative initiative of the American Heart Association and the Chinese Society of Cardiology. Detailed information about the design and methodology of the CCC-ACS has been described previously (9). In brief, the project included 241 PCI-capable hospitals from 30 provinces in China, using stratified sampling approach according to geographic region and economic status. First, 150 tertiary hospitals were enrolled from 2014 to 2015. Another 9 tertiary hospitals and 82 secondary hospitals were added from 2017 to 2018. The first 20 to 30 ACS patients admitted to tertiary hospitals and the first 10 to 20 ACS patients in secondary hospitals were recruited consecutively every month. They were identified using the principal discharge diagnosis. The CCC-ACS project was registered at ClinicalTrials.gov (NCT 02306616) and approved by the institutional review board of Beijing Anzhen Hospital, with a waiver for informed consent. A total of 94,623 inpatients with AMI, including 65,618 STEMI patients and 29,005 NSTEMI patients, were registered from November 2014 to December 2019. The flowchart of participant inclusion and exclusion is shown in Figure 1. Patients included in our study were stratified into inter-hospital transfer group (transferred from non-PCI hospital) vs. direct admission group (straight to PCI hospital) by the admission process to the registered hospitals.

### Data collection

Trained data abstractors entered the required clinical data from medical records to a web-based data collection platform (Oracle Clinical Remote Data Capture; Oracle Corporation, Redwood City, CA). Data elements included in this study were obtained from medical records. For quality reasons, third-party clinical research associates were entrusted to verify whether patient recruitment was in line with the plan of the project. About 5% of reported cases were randomly selected regularly, and validated with the original medical records by third-party research associates. Overall, the medical records abstraction achieved an accuracy of over 95% in this study.

### Study variables

Patient characteristics included demographics (age, sex, medical insurance), risk factors (hypertension, low-density lipoprotein cholesterol ≥ 70 mg/dL, diabetes mellitus, smoking, estimated glomerular filtration rate <60 mL⋅min^–1^⋅1.73 m^–2^), disease history (stroke/transient ischemic attacks, heart failure, and coronary heart disease), severe clinical conditions at admission (heart failure, cardiogenic shock, and cardiac arrest at admission), arrival at the first hospital by ambulance, acute medications in PCI-capable hospital [dual antiplatelet therapy (DAPT), glycoprotein IIb/IIIa inhibitors (GPIIb/IIIa), angiotensin-converting enzyme inhibitors/angiotensin receptor blockers (ACEI/ARBs), β-blockers and statins at arrival, and anticoagulant]. Details of definitions are shown in Supplementary Table 1.

Hospital characteristics included hospital level and regional economic level. Hospitals are graded into community health centers, secondary hospital, and tertiary hospital, based on the functionality, size, and specialization by National Health Commission of the People’s Republic of China. Secondary hospital are generally regional medical centers, and tertiary hospitals have large number of beds and provide comprehensive medical services. The 30 provinces in the mainland of China are divided into low, medium, or high economic levels according to the tertiles of the national gross domestic product. The map of regional economic level of China is shown in Supplementary Figure 1.

The relevant delays were defined as follows: treatment delay as time from symptom onset to primary PCI for STEMI and timely PCI for NSTEMI; door to balloon time as time from arrival at PCI-capable hospitals to primary PCI for STEMI; door to timely PCI as time from arrival at PCI-capable hospitals to timely PCI for NSTEMI.

Early revascularization included reperfusion therapy (fibrinolysis only and primary PCI only) and door-to-balloon within 90 min for STEMI, and timely PCI for NSTEMI. Timely PCI for NSTEMI was defined as a ≤24 h PCI from PCI hospital admission for high-risk patients and a ≤2 h PCI for very high-risk patients. The risk was classified using the risk stratification criteria proposed by the 2020 ESC guidelines for the management of NSTE-ACS (4).

In-hospital outcomes included major adverse cardiovascular events (MACE) and all-cause death, MACE was defined as a combination of all-cause death, recurrent myocardial infarction, stent thrombosis, and stroke during hospitalization.

### Statistical analysis

Categorical variables were described as frequencies and percentages, continuous variables were described as mean (standard deviation, SD) or median (interquartile range, IQR). The significance of differences in categorical variables was tested by chi-squared test. The unpaired *t*-test or Mann-Whitney U-test was used to test for statistically significant of differences between the mean or median, where appropriate.

The association between inter-hospital transfer vs. direct admission and in-hospital outcomes was assessed by Cox regression models. The time-point of MACE and mortality was the length of hospital stay. The proportional hazards assumption of each model was examined with log (–log[survival]) versus log (time) or Schoenfeld residuals test, and met. The multivariable Cox analyses adjusted the covariates in Table 1 plus year of admission, hospital characteristics, geographic area, symptom onset to PCI hospital admission, fibrinolysis (for STEMI only), and PCI (primary PCI, non-primary PCI and no PCI for STEMI; timely PCI, non-timely PCI and no PCI for NSTEMI).

**TABLE 1:** Characteristics of patients with AMI transferred from another hospital or directly admitted.

	AMI		*P* value	STEMI		*P* value	NSTEMI		*P* value
					
	Inter-hospital transfer (*N* = 10,141)	Direct admission (*N* = 18,864)		Inter-hospital transfer (*N* = 30,829)	Direct admission (*N* = 34,789)		Inter-hospital transfer value	Direct admission (*N* = 53,653)	
Age, year	61.9 (12.5)	63.9 (12.7)	<0.001	61.3 (12.6)	62.8 (12.8)	<0.001	63.6 (12.1)	66.1 (12.4)	<0.001
Female	9518 (23.2)	13,839 (25.8)	<0.001	6699 (21.7)	8001 (23.0)	<0.001	2819 (27.8)	5838 (30.9)	<0.001
**Medical insurance**			<0.001			<0.001			<0.001
High reimbursement	18,483 (45.1)	33,973 (63.3)		13,501 (43.8)	21,622 (62.2)		4982 (49.1)	12,351 (65.5)	
Medium reimbursement	12,172 (29.7)	10,290 (19.2)		9586 (31.1)	6714 (19.3)		2586 (25.5)	3576 (19.0)	
Low reimbursement	10,315 (25.2)	9390 (17.5)		7742 (25.1)	6453 (18.5)		2573 (25.4)	2937 (15.6)	
**Risk factor**									
Hypertension	24,896 (60.8)	36,742 (68.5)	<0.001	18,059 (58.6)	22,637 (65.1)	<0.001	6837 (67.4)	14,105 (74.8)	<0.001
LDL-C ≥ 70mg/dL	34,736 (84.8)	46,140 (86.0)	<0.001	26,311 (85.3)	30,491 (87.6)	<0.001	8425 (83.1)	15,649 (83.0)	0.793
Diabetes mellitus	10,644 (26.0)	15,798 (29.4)	<0.001	7594 (24.6)	9391 (27.0)	<0.001	3050 (30.1)	6407 (34.0)	<0.001
Smoking	18,967 (46.3)	21,140 (39.4)	<0.001	14,821 (48.1)	14,913 (42.9)	<0.001	4146 (40.9)	6227 (33.0)	<0.001
eGFR < 60 mL⋅min^–1^⋅1.73 m^–2^	7435 (18.1)	10,994 (20.5)	<0.001	5345 (17.3)	6429 (18.5)	<0.001	2090 (20.6)	4565 (24.2)	<0.001
**Disease history**									
CHD	2606 (6.4)	6743 (12.6)	<0.001	1538 (5.0)	3093 (8.9)	<0.001	1068 (10.5)	3650 (19.3)	<0.001
Heart failure	421 (1.0)	1434 (2.7)	<0.001	205(0.7)	421 (1.2)	<0.001	216(2.1)	1013 (5.4)	<0.001
Stroke/TIA	3360 (8.2)	4924 (9.2)	<0.001	2371 (7.7)	2783 (8.0)	0.142	989 (9.8)	2141 (11.3)	<0.001
**Severe clinical condition at admission**									
Heart failure	3080 (7.5)	3687 (6.9)	<0.001	2276 (7.4)	2064 (5.9)	<0.001	804(7.9)	1623 (8.6)	0.048
Cardiogenic shock	1304 (3.2)	1470 (2.7)	<0.001	1142 (3.7)	1212 (3.5)	0.130	162 (1.6)	258 (1.4)	0.118
Cardiac arrest	781 (1.9)	762 (1.4)	<0.001	691(2.2)	628 (1.8)	<0.001	90 (0.9)	134 (0.7)	0.100
Arriving the first hospitals by ambulance	2198 (5.4)	4839 (9.0)	<0.001	1865(6.0)	3956 (11.4)	<0.001	333(3.3)	883 (4.7)	<0.001
**Medications**									
DAPT at arrival	38,972 (95.1)	49,593 (92.4)	<0.001	29,521 (95.8)	32,755 (94.2)	<0.001	9451 (93.2)	16,838 (89.3)	<0.001
Aspirin	39,329 (96.0)	50,707 (94.5)	<0.001	29,745 (96.5)	33,295 (95.7)	<0.001	9584 (94.5)	17,412 (92.3)	<0.001
P2Y12 inhibitors	39,690 (96.9)	50,802 (94.7)	<0.001	29,985 (97.3)	33,264 (95.6)	<0.001	9705 (95.7)	17,538 (93.0)	<0.001
ACEIs/ARBs at arrival	20,017 (48.9)	25,380 (47.3)	<0.001	14,720 (47.7)	15,782 (45.4)	<0.001	5297 (52.2)	9598 (50.9)	0.028
β-Blockers at arrival	23,374 (57.1)	29,558 (55.1)	<0.001	17,281 (56.1)	18,329 (52.7)	<0.001	6093 (60.1)	11,229 (59.5)	0.357
Statins at arrival	38,777 (94.6)	50,050 (93.3)	<0.001	29,215 (94.8)	32,486 (93.4)	<0.001	9562 (94.3)	17,564 (93.1)	<0.001
GPIIb/IIIa at arrival	13,207 (32.2)	15,593 (29.1)	<0.001	10,986 (35.6)	12,209 (35.1)	0.148	2221 (21.9)	3384 (17.9)	<0.001
Anticoagulant	30,518 (74.5)	40,536 (75.6)	<0.001	23,131 (75.0)	26,373 (75.8)	0.021	7387 (72.8)	14,163 (75.1)	<0.001

Values are mean (standard deviation, SD), or n (%).

ACEIs/ARBs, angiotensin-converting enzyme inhibitors/angiotensin receptor blockers; AMI, acute myocardial infarction; CHD, coronary heart disease; DAPT, dual antiplatelet therapy; eGFR, estimated glomerular filtration rate; LDL-C, low-density lipoprotein cholesterol; GPIIb/IIIa, glycoprotein IIb/IIIa inhibitors; NSTEMI, non-ST-segment elevation myocardial infarction; STEMI, ST-segment elevation myocardial infarction; and TIA, transient ischemic attacks.

The inverse probability of treatment weighting (IPTW) analyses were conducted to further control of confounding, the propensity score (PS) was calculated in a logistic regression model included covariates in Table 1 and AMI type (STEMI and NSTEMI), year of admission, hospital characteristics, geographic area (North, northeast, south, southwest, east, northwest and central China), time from symptom onset to PCI hospital admission. The inverse of the PS and the inverse of 1 minus the PS, were calculated as the weight of each patient in inter-hospital transfer and direct admission groups, respectively. To make the sum of weights close to the size of the original sample, a stabilized IPTW was calculated (10, 11). We divided the IPTW weights by the proportion of direct admission or transferred (as part of the whole completed data) for directly admitted and transferred patients, respectively. Group differences were assessed by standardized mean differences (SMD), SMD < 10.0% for these included variables indicated a relatively small imbalance (12).

Sensitivity analyses were carried out after excluding patients with missing values for any variable used in the analyses. Missing rates of variables and the strategies for managing missing data are described in Supplementary Table 2. R (version 3.6.2) and Stata 14.0 (Stata, College Station, TX, USA) were used for data analyses. Two-sided *P* < 0.05 were considered statistically significant.

## Results

A total of 94,623 AMI patients were included in this study. Overall, 43.3% of patients hospitalized with AMI were transferred. The proportion was higher in patients with STEMI than those with NSTEMI (47.0% vs. 35.0%, *P* < 0.001). Compared with patients directly admitted to PCI-capable hospitals, patients transferred from other hospitals were younger, more likely to present with severe clinical conditions, had a higher rate of low-reimbursement medical insurance, and higher rates acute medications at arrival (Table 1).

### Variations in proportions of patients transferred between hospitals

**FIGURE 2:**
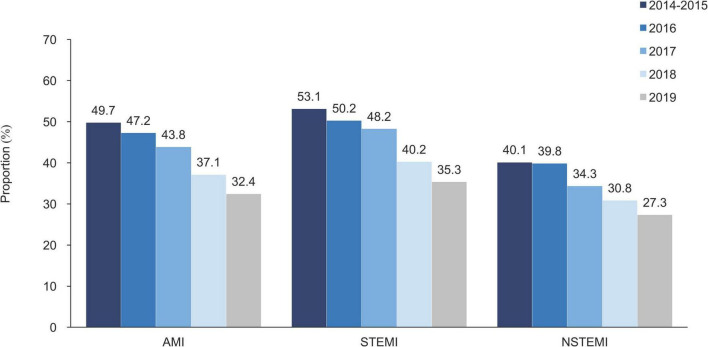
Proportion of inter-hospital transfer in patients with AMI, by the year of admission. AMI, acute myocardial infarction; NSTEMI, non-ST-segment elevation myocardial infarction; and STEMI, ST-segment elevation myocardial infarction.

**FIGURE 3:**
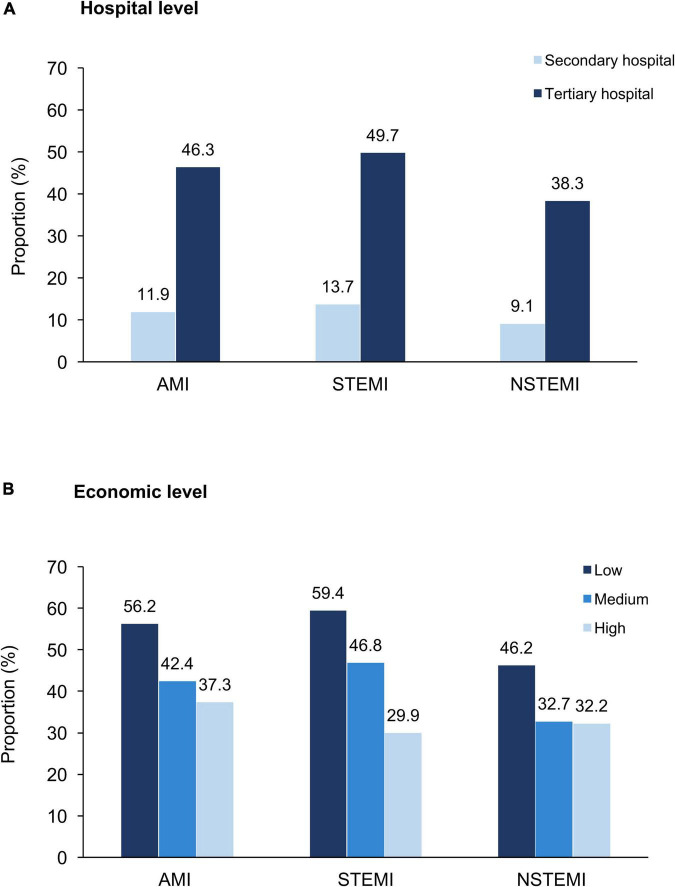
Proportion of inter-hospital transfer in patients with AMI, by hospital characteristics. **(A)** Hospital level and **(B)** Economic level. AMI, acute myocardial infarction; NSTEMI, non-ST-segment elevation myocardial infarction; and STEMI, ST-segment elevation myocardial infarction.

From 2014 to 2019, the proportion of transferred patients decreased significantly, falling from 53.1 to 35.3% for STEMI and 40.1% to 27.3% for NSTEMI (Figure 2). Hospitals with higher proportion of patients transferred admitted more patients with STEMI than NSTEMI (Supplementary Figure 2). The proportions of transferred patients with AMI varied considerably across hospital levels and economic levels. Tertiary hospitals had higher rates of inter-hospital transfer than secondary hospitals (46.3% vs. 11.9%), and hospitals in low-economic regions had higher rates than more affluent areas (56.2% vs. 37.3%) (Figure 3).

### Treatment delay

Transferred patients had longer delays from symptom onset to early revascularization for both STEMI [median (IQR): 6.5 h (4.0–11.2) vs. 4.5 h (2.7–8.5), *P* < 0.001] and NSTEMI [median (IQR):16.3 h (8.7–33.0) vs.11.9 h (5.7–25.5), *P* < 0.001]. However, they had a significantly shorter door to balloon time for STEMI [median (IQR): 0.8 h (0.4–1.5) vs. 1.0 h [0.6–1.8], *P* < 0.001] or door to timely PCI for NSTEMI [median (IQR): 2.8 h (1.1–14.2) vs. 3.7 h (1.5–10.8), *P* = 0.002] than directly admitted patients (Supplementary Table 3).

### Early revascularization

The disparities in early revascularization between transferred and directly admitted patients were shown in Table 2. For STEMI, reperfusion therapies were performed in 65.2% of directly admitted patients and 57.8% of transferred patients. Of the reperfusion strategies for STEMI, patients transferred from non-PCI-capable hospitals were more likely to receive fibrinolysis (10.9% vs. 4.8%, *P* < 0.001) and less likely to receive primary PCI (46.9% vs. 60.4%, *P* < 0.001). Door-to-balloon time of <90 min was achieved in a larger proportion of inter-hospital transferred patients compared to those of direct admissions for patients who received primary PCI (75.2% vs. 69.9%, *P* < 0.001). For NSTEMI, patients transferred between hospitals were less likely to receive timely PCI than directly admitted patients (34.7% vs. 41.1%, *P* < 0.001). Similar results were observed in the IPTW sample (Table 2).

**TABLE 2:** Early revascularization of patients with AMI who were transferred from another hospital and directly admitted.

	Entire sample			IPTW sample		
		
	Inter-hospital transfer	Direct admission	*P* value	Inter-hospital transfer	Direct admission	*P* value
**STEMI**						
Reperfusion	57.8 (17,817/30,829)	65.2 (22,677/34,789)	<0.001	67.1 (15,014/22,382)	70.6 (20,049/28,414)	<0.001
Fibrinolysis only	10.9 (3361/30,829)	4.8 (1665/34,789)	<0.001	10.9 (2446/22,382)	5.2 (1480/28,414)	<0.001
Primary PCI only	46.9 (14,456/30,829)	60.4 (21,012/34,789)	<0.001	56.2 (12,568/22,382)	65.4 (18,569/28,414)	<0.001
DTB within 90min*	75.2 (8842/11,763)	69.9 (12,238/17,514)	<0.001	77.1(8235/10,687)	70.9 (11,427/16,112)	<0.001
**NSTEMI**						
Timely PCI^†^	34.7 (1900/5478)	41.1 (3401/8271)	<0.001	41.3 (1447/3504)	45.6 (2693/5908)	<0.001

*The denominator was STEMI patients who received primary PCI with DTB time available (included STEMI patients who received primary PCI and excluded patients without DTB times), the number of denominator was 29,277 (35,468 minus 6191) in entire sample and 26,799 (31,137 minus 4338) in IPTW sample. ^†^The denominator was NSTEMI patients who received PCI with PCI time available (included NSTEMI patients who received PCI and excluded those have no information on the timing of PCI), the number of denominator was 13,749 (17,554 minus 3805) in entire sample and 9412 (11,349 minus 1937) in IPTW sample.

DTB, door-to-balloon; NSTEMI, non-ST-segment elevation myocardial infarction; PCI, percutaneous coronary intervention; and STEMI, ST-segment elevation myocardial infarction.

### In-hospital outcomes

The median time-point was 9 days for MACE and mortality (Table 3). In-hospital outcomes were shown in Table 3. Lower rates of in-hospital outcomes were observed in the inter-hospital transfer group. However, after multivariable adjustment by Cox regression, there were no significant differences in in-hospital MACE and all-cause death between the inter-hospital transfer and direct admission groups. The IPTW analysis formed by all covariates included in the propensity analysis also resulted in between-group balance on basic characteristics (Supplementary Table 4), and revealed similar results to the entire samples. The hazard ratios and 95% confidence intervals in the IPTW models were 0.87 (0.75–1.01) and 0.87 (0.73–1.03) for MACE and mortality, respectively, in patients with STEMI, and 1.02 (0.71–1.48) and 0.98 (0.70–1.35), respectively, in patients with NSTEMI (Table 3).

**TABLE 3:** The association between in-hospital outcomes and inter-hospital transfer vs. direct admission in patients with STEMI and NSTEMI.

	Entire sample				IPTW sample			
		
	Inter-hospital transfer	Direct admission	HR (95%CI)*	*P* value	Inter-hospital transfer	Direct admission	HR (95%CI)	*P* value
**STEMI**								
MACE	2.3 (706/30,829)	3.4 (1187/34,789)	0.90 (0.80–1.02)	0.100	2.3 (523/22,382)	3.3 (944/28,414)	0.87 (0.75–1.01)	0.060
Death	1.6 (499/30,829)	2.5 (866/34,789)	0.93 (0.82–1.07)	0.310	1.7 (381/22,382)	2.5 (699/28,414)	0.87 (0.73–1.03)	0.106
**NSTEMI**								
MACE	1.9 (194/10,141)	3.0 (558/18,864)	1.05 (0.82–1.34)	0.707	1.7 (104/5947)	2.8 (365/12,938)	1.02 (0.71–1.48)	0.908
Death	1.3 (127/10,141)	1.9 (356/18,864)	1.17 (0.88–1.57)	0.282	1.2 (73/5947)	1.7 (220/12,938)	0.98 (0.70–1.35)	0.879

The median time-point was 9 days for both MACE and mortality. *Adjusted for age, sex, medical insurance, risk factors (hypertension, LDL-C ≥ 70 mg/dL, eGFR < 60 mLmin^–1^1.73 m^–2^, diabetes mellitus, and smoking), disease history (CHD, heart failure, and stroke/TIA), severe clinical condition at admission (heart failure, cardiogenic shock, cardiac arrest), time from symptom onset to PCI hospital admission, ambulance, DAPT, GPIIb/IIIa, ACEI/ARBs, β-blockers and statins at arrival, anticoagulant, year of admission, characteristics of hospital (hospital level, economic level), geographical area, fibrinolysis (only for STEMI), and PCI (primary PCI, non-primary PCI and no PCI for STEMI; timely PCI, non-timely PCI and no PCI for NSTEMI).

CI, confidence interval; HR, hazard ratio; IPTW, inverse probability of treatment weighting; MACE, major adverse cardiovascular events; NSTEMI, non-ST-segment elevation myocardial infarction; and STEMI, ST-segment elevation myocardial infarction.

### Sensitivity analyses

The treatment delay, early revascularization, and in-hospital outcomes were compared for transferred and directly admitted patients, after excluding all patients with missing values for any variable used in the analyses. Similar results were found, as shown in Supplementary Tables 5–7.

## Discussion

Through this real-world nationwide registry, we provided a comprehensive and contemporary overview of the inter-hospital transfer of patients with AMI in China. On average, over 40% of AMI patients were still transferred from non-PCI-capable hospitals to PCI-capable hospitals during 2014 to 2019, although a decreasing trend in the rate of inter-hospital transfer was observed. We also found a significant variation in the rates of inter-hospital transfer among different hospital levels and economic levels. Transferred patients had lower rates of early revascularization and longer treatment delay. However, the differences in in-hospital outcomes were not significant after adjustment for confounding factors.

The major initiative to improve the prognosis of AMI is to minimize the time from symptom onset to reperfusion. Myocardial cell death begins as early as 20 min after coronary artery occlusion, and the time from symptom onset to balloon is a significant determinant of myocardial damage and risk of mortality (13, 14). Direct admission to a PCI-capable hospital for PCI as soon as possible is therefore recommended by Chinese Society of Cardiology, American Heart Association and European Society of Cardiology for patients with AMI (2–4, 15, 16). However, interventional facilities are limited or unevenly distributed in practice, and some patients first present at a non-PCI-capable hospital (17, 18).

Previous studies in western countries reported that approximately 22%–40% of STEMI patients were initially admitted to a referring facility and subsequently transferred to PCI-capable facilities for primary PCI (6, 19–21). However, in our study, 47.0% of STEMI patients were transferred from another hospital. This proportion was even higher than the 45.2% reported in the CREDO-Kyoto (Coronary Revascularization Demonstrating Outcome Study in Kyoto) AMI registry that took place between 2005 and 2007 in Japan (22). In addition, a considerable variation in the proportion of inter-hospital transfer among hospitals were observed in our study, particularly hospitals in regions with different levels of prosperity, and at different academic levels. Fine medical resource and experienced experts usually centers in big cities or developed areas (23). The limited availability of hospitals with revascularization capacity and interventionalists qualified for PCI in areas of low economic level may be related to the high rate of inter-hospital transfer to the PCI-capable hospitals of these areas. Moreover, among all PCI-capable hospitals in this study, the proportion of transferred patients in tertiary hospitals was higher than that of secondary hospitals. The difference in inter-hospital transfer rates among hospitals levels can be partly attribute to the special functions of different grades of hospitals. Secondary hospitals are regional hospitals that provide medical health services across several communities, and receive referrals from patients in community hospitals. Tertiary hospitals provide more comprehensive medical services, and often accept referrals from secondary hospitals. And beyond that, the lack of effective hierarchical structure of the medical system in China may have aggravated this gap (24). In order to rationally allocate medical resources and promote equalization of basic medical and health services, the General Office of the State Council issued the Guiding Opinions on Promoting the Construction of the Hierarchical Medical System on September 8, 2015. With the progression of the national diagnosis and treatment system, the PCI capacity of secondary hospitals has been gradually improved. The decreasing trend found in the rate of inter-hospital transfer from 2015 to 2019 may indicate an improvement in medical services for patients with AMI. However, additional measures such as increasing the allocation of PCI hospitals and improving the revascularization capacity of secondary hospitals are still needed to narrow the gap with western countries and minimize the inequality across regions.

Current guidelines recommend that patients with STEMI who were initially admitted to a non-PCI-capable hospital should be transferred immediately to a PCI-capable hospital (2, 3). We found that inter-hospital transfer was associated with a 2.0 h additional time from symptom onset to primary PCI; however, there was a slightly shorter door-to-balloon time in patients who were transferred than those who arrived directly at PCI-capable hospitals. Similar results in Polish registry of Acute Coronary Syndromes also indicated that transferred patients had a shorter admission-to-PCI time than patients admitted directly (6). It is not at all surprising that door-to-balloon time would be shorted in transferred patients following arrival to the PCI capable hospital because primary evaluation in the first hospital would help determine whether the patient was diagnosed with STEMI prior to being transfer to the PCI-capable hospitals, and the cath teams are often already waiting at the hospital for those patients in anticipation of their arrival, rather than needing to come in from home. The median door-to-balloon time of 1.0 h and 0.8 h for directly admitted and transferred STEMI patients in our study was much lower than that of 115 min found in the China STEMI Care Project Phase 1 in 2012 (25). The improvement of door to balloon time may be the result of the construction of the hierarchical medical system in 2015 and the officially established of the chest pain center (CPC) accreditation in 2016 (26). The median door-to-balloon time in our study for STEMI patients following inter-hospital transfer was within the guideline recommendations, indicating that the time from symptom onset to arrival at PCI-capable hospitals might be the major source of delay in China (2, 15). However, the door-to-balloon time in STEMI was still far from optimal, indicating that there is still room for further improvement through close inter-hospital connection and in-hospital process optimization. For patients with NSTEMI first arrived at a non-PCI center, the time range is broader. PCI should be done within 24 h if possible, but patients with very-high-risk features require immediate invasive strategies (1). In spite of longer delay from symptom onset to timely PCI, the early risk stratification before inter-hospital transfer may be responsible for the shorter door to timely PCI time for transferred group than direct admission.

We observed that transferred patients were less likely to receive early revascularization than directly admitted. For STEMI, the reperfusion rates were approximately 60% in both groups, which was much lower than the rates in western countries (27, 28). The prolonged treatment delays and limited capacity of revascularization services were considered as the major contributing factors of the low performance rates of reperfusion therapy in China (29). Of the specific reperfusion strategies, primary PCI is preferred over an initial fibrinolysis strategy and has a significant lower risk of bleeding complications, but fibrinolysis is recommended for STEMI patients if primary PCI is not feasible within 120 min of the first medical contact (3). The lower rate of primary PCI and higher fibrinolysis rate of inter-hospital transfer group is therefore consistent with their longer delays compared with direct admitted patients. For NSTEMI, a timely performance of PCI would reduce ischemic risk. Similar with STEMI, data from the present study showed significantly less likely of timely PCI (34.7% vs. 41.1%) with transferred patients compared with those admitted directly.

Despite the faster time to treatment and higher rate of reperfusion among directly admitted patients, there were no significant differences between the two groups in in-hospital outcomes after multivariable adjustment. These results were similar to those from the RACE registry in North Carolina, which also compared direct admission to PCI hospitals with inter-hospital transfer and found that inter-hospital transfer was not related to adverse in-hospital outcomes after multivariable adjustment (5). Similarly, the Harmonizing Outcomes with Revascularization and Stent in Acute Myocardial Infarction study found that 30-day and 1-year clinical outcomes were also comparable in patients with STEMI directly admitted or transferred for primary PCI (7). The following pathophysiological mechanisms seem to offer a plausible explanation for these findings. Acute coronary obstruction precipitates ischemia, which is still reversible initially and associated with minimal myocardial necrosis. Without coronary reperfusion, myonecrosis will develop rapidly early on, and gradually slow down as damage becomes more complete (13). During the first 2 to 3 h after symptom onset, there are significant benefits of reperfusion, but after that, the benefit decreases with time (30). The median time from symptoms to balloon in our study far exceeded this time window. Thus, despite higher rate of reperfusion in directly admitted patients, there was no significant difference in in-hospital outcomes. Therefore, the time delay from presentation to revascularization deserves more attention in order to improve AMI care. Nonetheless, the relatively lower rates of MACE and death in the transferred group may also be explained by the positive pre-selection in this group. Compared with the directly admitted patients, the transferred patients may suffer longer delay before interventional therapy, which can lead to a higher out-of-hospital death rate prior to PCI-capable hospital and a lower in-hospital risk of adverse events.

Our findings suggest that there has been a dramatic improvement in the access to medical services for patients with AMI over the past few years. Whereas, additional measures are needed to improve the management of AMI. The government should attach great importance to improving the medical security system and increase investment in the medical and health field, so that more patients with AMI, especially those in areas with low economic level, can receive timely treatment. Direct admission to PCI-capable hospitals is the preferred strategy for patients with AMI, enabling faster treatment and higher rate of early revascularization. Yet, more great effort should be made to shorten the time interval from symptom onset to revascularization, which seems to substantially impact the benefit of early revascularization. This requires the awareness of the suspected ischemic symptoms of the public in order to call the common emergency number for help or be sent directly to PCI hospitals by other family members immediately. Furthermore, telemedicine should be implemented to facilitate patient transfer. It was reported that the coverage rate of tele-electrocardiography in tertiary hospitals is less than 40% (31). Steps can be taken to increase the utilization of tele-electrocardiography as one measure to improve outcomes, as the patient could be directly transferred to PCI center. But if direct admission to PCI hospital is not feasible, rapid access to a non-interventional hospital for professional diagnosis and timely treatments, and then transfer to a PCI hospital when necessary is also an alternative. Since a large proportion of AMI were transferred, an efficient local transfer network from non-PCI hospitals to PCI hospitals should be essential to shorten the time delay (32).

Our study had several limitations. First, it included only patients who were admitted to a hospital that was part of the CCC-ACS project. Patients who died before arrival were not included in this analysis, which may lead to a survival bias. Second, we only accessed the in-hospital MACE and mortality. Future studies that track patients after discharge will help to assess the difference in long-term outcomes between direct admission and inter-hospital transfer patients. Third, it appears a delay in initial presentation to a non-PCI hospital importantly influences the longer symptom-to-revascularization time, however, reliable time information in the first hospital was not available for the transferred patients. Even leaving aside the time information in non-PCI hospitals, the data of treatment delay was not available of about 20% of patients. Fourth, only 5% of the records were audited by third-party research associates due to the large sample size, although the randomly selected records were expected to be representative. Finally, we imputed the missing values for some variables. Poorly imputation of missing value can damage the quality and reliability of the results. We excluded the patients with missing values for any variable used in our study, and found similar results in the sensitivity analyses, suggesting the results of our study are credible.

## Conclusion

A large proportion of patients hospitalized with AMI are transferred from non-PCI hospitals in China, especially in low-economic regions. There are significant differences in treatment delay, and use of early revascularization between transferred patients and directly admitted patients. National strategies are needed to promote the recommended revascularization strategies and minimize the inequality in management of AMI.

## Data availability statement

The original contributions presented in this study are included in the article/Supplementary material, further inquiries can be directed to the corresponding authors.

## Author contributions

DZ and JiL were conceived and designed the study. DZ, JiL, YH, JuL, and NY were collected and interpreted the data. DH was analyzed the data and prepared the first draft of the manuscript. All authors critically revised manuscript for important intellectual content and read and approved the final manuscript.

## References

[1] Reed G Rossi J Cannon C. Acute myocardial infarction. *Lancet.* (2017) 389:197–210. 10.1016/S0140-673630677-8 27502078

[2] O’Gara P Kushner F Ascheim D Casey D Jr. Chung M de Lemos J 2013 ACCF/AHA guideline for the management of ST-elevation myocardial infarction: executive summary: a report of the American college of cardiology foundation/American heart association task force on practice guidelines. *Circulation.* (2013) 127:529–55. 10.1161/CIR.0b013e3182742c84 23247303

[3] Ibanez B James S Agewall S Antunes M Bucciarelli-Ducci C Bueno H 2017 ESC guidelines for the management of acute myocardial infarction in patients presenting with ST-segment elevation: the task force for the management of acute myocardial infarction in patients presenting with ST-segment elevation of the European society of cardiology (ESC). *Eur Heart J.* (2018) 39:119–77. 10.1093/eurheartj/ehx393 28886621

[4] Collet J Thiele H Barbato E Barthelemy O Bauersachs J Bhatt D 2020 ESC guidelines for the management of acute coronary syndromes in patients presenting without persistent ST-segment elevation. *Eur Heart J.* (2021) 42:1289–367. 10.1093/eurheartj/ehaa575 32860058

[5] Fosbol E Granger C Jollis J Monk L Lin L Lytle B The impact of a statewide pre-hospital STEMI strategy to bypass hospitals without percutaneous coronary intervention capability on treatment times. *Circulation.* (2013) 127:604–12. 10.1161/CIRCULATIONAHA.112.118463 23275382

[6] Kawecki D Gierlotka M Morawiec B Hawranek M Tajstra M Skrzypek M Direct admission versus interhospital transfer for primary percutaneous coronary intervention in ST-segment elevation myocardial infarction. *JACC Cardiovasc Interv.* (2017) 10:438–47. 10.1016/j.jcin.2016.11.028 28216215

[7] Wohrle J Desaga M Metzger C Huber K Suryapranata H Guetta V Impact of transfer for primary percutaneous coronary intervention on survival and clinical outcomes (from the HORIZONS-AMI trial). *Am J Cardiol.* (2010) 106:1218–24. 10.1016/j.amjcard.2010.06.049 21029816

[8] Kim B Cha K Park M Choi J Yun E Park J The impact of transferring patients with ST-segment elevation myocardial infarction to percutaneous coronary intervention-capable hospitals on clinical outcomes. *Cardiol J.* (2016) 23:289–95. 10.5603/CJ.a2016.0003 26779970

[9] Hao Y Liu J Liu J Smith S Jr. Huo Y Fonarow G Rationale and design of the improving care for cardiovascular disease in China (CCC) project: a national effort to prompt quality enhancement for acute coronary syndrome. *Am Heart J.* (2016) 179:107–15. 10.1016/j.ahj.2016.06.005 27595685

[10] Cole S Hernan M. Adjusted survival curves with inverse probability weights. *Comput Methods Programs Biomed.* (2004) 75:45–9. 10.1016/j.cmpb.2003.10.004 15158046

[11] Xu S Ross C Raebel M Shetterly S Blanchette C Smith D. Use of stabilized inverse propensity scores as weights to directly estimate relative risk and its confidence intervals. *Value Health.* (2010) 13:273–7. 10.1111/j.1524-4733.2009.00671.x 19912596 PMC4351790

[12] Austin P. Using the standardized difference to compare the prevalence of a binary variable between two groups in observational research. *Commun Simulat Comput.* (2009) 38:1228–34. 10.1080/03610910902859574

[13] Nallamothu B Bradley E Krumholz H. Time to treatment in primary percutaneous coronary intervention. *N Engl J Med.* (2007) 357:1631–8. 10.1056/NEJMra065985 17942875

[14] Terkelsen C Sorensen J Maeng M Jensen L Tilsted H Trautner S System delay and mortality among patients with STEMI treated with primary percutaneous coronary intervention. *JAMA.* (2010) 304:763–71. 10.1001/jama.2010.1139 20716739

[15] Chinese Society of Cardiology of Chinese Medical Association, Editorial Board of Chinese Journal of Cardiology. [2019 Chinese society of cardiology (CSC) guidelines for the diagnosis and management of patients with ST-segment elevation myocardial infarction]. *Zhonghua Xin Xue Guan Bing Za Zhi.* (2019) 47:766–83. 10.3760/cma.j.issn.0253-3758.2019.10.003 31648459

[16] Chinese Society of Cardiology of Chinese Medical Association, Editorial Board of Chinese Journal of Cardiology. [2016 Chinese society of cardiology (CSC) guidelines for the diagnosis and management of patients with non-ST-segment elevation acute coronary syndrom]. *Zhonghua Xin Xue Guan Bing Za Zhi.* (2017) 45:5. 10.3760/cma.j.issn.0253-3758.2019.10.003 31648459

[17] Zhang Q Zhang R Qiu J Zhang J Wang X Jiang L One-year clinical outcome of interventionalist- versus patient-transfer strategies for primary percutaneous coronary intervention in patients with acute ST-segment elevation myocardial infarction: results from the REVERSE-STEMI study. *Circ Cardiovasc Qual Outcomes.* (2011) 4:355–62. 10.1161/CIRCOUTCOMES.110.958785 21521833

[18] Concannon T Nelson J Goetz J Griffith J. A percutaneous coronary intervention lab in every hospital? *Circ Cardiovasc Qual Outcomes.* (2012) 5:14–20. 10.1161/CIRCOUTCOMES.111.963868 22147882 PMC4440579

[19] Rathod K Jain A Firoozi S Lim P Boyle R Nevett J Outcome of inter-hospital transfer versus direct admission for primary percutaneous coronary intervention: an observational study of 25,315 patients with ST-elevation myocardial infarction from the London heart attack group. *Eur Heart J Acute Cardiovasc Care.* (2020) 9:948–57. 10.1177/2048872619882340 32193943

[20] Dieker H Liem S El Aidi H van Grunsven P Aengevaeren W Brouwer M Pre-hospital triage for primary angioplasty: direct referral to the intervention center versus interhospital transport. *JACC Cardiovasc Interv.* (2010) 3:705–11. 10.1016/j.jcin.2010.04.010 20650431

[21] Liebetrau C Szardien S Rixe J Woelken M Rolf A Bauer T Direct admission versus transfer of AMI patients for primary PCI. *Clin Res Cardiol.* (2011) 100:217–25. 10.1007/s00392-010-0231-x 20857125

[22] Nakatsuma K Shiomi H Morimoto T Furukawa Y Nakagawa Y Ando K Inter-facility transfer vs. direct admission of patients with ST-segment elevation acute myocardial infarction undergoing primary percutaneous coronary intervention. *Circ J.* (2016) 80:1764–72. 10.1253/circj.CJ-16-0204 27350014

[23] Zhao P Li S Liu D. Unequable spatial accessibility to hospitals in developing megacities: new evidence from Beijing. *Health Place.* (2020) 65:102406. 10.1016/j.healthplace.2020.102406 32877867 PMC7456595

[24] Du X Patel A Anderson C Dong J Ma C. Epidemiology of cardiovascular disease in china and opportunities for improvement: JACC international. *J Am Coll Cardiol.* (2019) 73:3135–47. 10.1016/j.jacc.2019.04.036 31221263

[25] Zhang Y Tian Y Dong P Xu Y Yu B Li H Treatment delay and reperfusion management of acute ST-segment elevation myocardial infarction-analysis of the China STEMI care project phase 1 (CSCAP-1). *QJM.* (2020) 2020:186. 10.1093/qjmed/hcaa186 32569364

[26] Sun P Li J Fang W Su X Yu B Wang Y Effectiveness of chest pain centre accreditation on the management of acute coronary syndrome: a retrospective study using a national database. *BMJ Qual Saf.* (2021) 30:867–75. 10.1136/bmjqs-2020-011491 33443197

[27] Schiele F Hochadel M Tubaro M Meneveau N Wojakowski W Gierlotka M Reperfusion strategy in Europe: temporal trends in performance measures for reperfusion therapy in ST-elevation myocardial infarction. *Eur Heart J.* (2010) 31:2614–24. 10.1093/eurheartj/ehq305 20805111

[28] Masoudi F Ponirakis A de Lemos J Jollis J Kremers M Messenger J Trends in U.S. cardiovascular care: 2016 report from 4 ACC national cardiovascular data registries. *J Am Coll Cardiol.* (2017) 69:1427–50. 10.1016/j.jacc.2016.12.005 28025065

[29] Ranasinghe I Rong Y Du X Wang Y Gao R Patel A System barriers to the evidence-based care of acute coronary syndrome patients in China: qualitative analysis. *Circ Cardiovasc Qual Outcomes.* (2014) 7:209–16. 10.1161/CIRCOUTCOMES.113.000527 24619324

[30] Gersh B Stone G White H Holmes D Jr. Pharmacological facilitation of primary percutaneous coronary intervention for acute myocardial infarction: is the slope of the curve the shape of the future? *JAMA.* (2005) 293:979–86. 10.1001/jama.293.8.979 15728169

[31] Cui F Ma Q He X Zhai Y Zhao J Chen B Implementation and application of telemedicine in china: cross-sectional study. *JMIR Mhealth Uhealth.* (2020) 8:e18426. 10.2196/18426 33095175 PMC7647817

[32] Zhang Y Yu B Han Y Wang J Yang L Wan Z Protocol of the China ST-segment elevation myocardial infarction (STEMI) care project (CSCAP): a 10-year project to improve quality of care by building up a regional STEMI care network. *BMJ Open.* (2019) 9:e026362. 10.1136/bmjopen-2018-026362 31320346 PMC6661651

