# Supplementary material for: Inter-hospital transfer in patients with acute myocardial infarction in China: Findings from the improving care for cardiovascular disease in China-acute coronary syndrome project
Source: Front Cardiovasc Med. 2022 Dec 8;9:1064690. doi: 10.3389/fcvm.2022.1064690 (PMC9773877; doi:10.3389/fcvm.2022.1064690)
Supplement: Supplementary file 1 [file Data_Sheet_1.PDF]

Supplemental Material

Supplementary Table 1. Definition of baseline variables

Supplementary Table 2. Missing rates of study variables and management of missing data (variables without any missing data are not listed in the table)

Supplementary Table 3. The median delay in receiving early revascularization of patients with STEMI and NSTEMI transferred from another hospital and directly admitted

Supplementary Table 4. Characteristics of inter-hospital transfer vs. direct admission in patients with AMI in the inverse probability of treatment-weighted sample

Supplementary Table 5. The median delay in receiving early revascularization patients with STEMI and NSTEMI who were transferred from another hospital and directly admitted, after excluding missing data

Supplementary Table 6. Early revascularization of patients with STEMI and NSTEMI who were transferred from another hospital and directly admitted, after excluding missing data

Supplementary Table 7. The association between in-hospital outcomes and inter-hospital transfer vs. direct admission in patients with STEMI and NSTEMI, after excluding missing data

Supplementary Table 8. Investigators of CCC-ACS project

Supplementary Figure 1. The map of regional economic level of China

Supplementary Figure 2. Proportion of patients with STEMI and NSTEMI in hospitals with different inter-hospital transfer rates

Supplementary Table 1. Definition of baseline variables

	**Definition**
**Age**	Years after birth
**Female**	Female
**Medical insurance**	Insurance with full reimbursement, basic medical insurance for urban employees, basic medical insurance for urban unemployed residents and commercial insurance were combined as medical insurance with high reimbursement. New rural cooperative medical insurance was defined as medical insurance with medium reimbursement. No medical insurance, special assistance for the poor and other types of medical insurance were defined as medical insurance with low reimbursement
**Risk factor**	
Hypertension	Previously diagnosed with hypertension, receiving antihypertensive therapy, or having a systolic blood pressure ≥ 140 mm Hg or diastolic blood pressure ≥ 90 mmHg at admission
LDL-C ≥ 70mg/dL	Serum LDL-C level ≥ 1.8 mmol/L (70 mg/dL)
Diabetes mellitus	Previous diagnosis of diabetes mellitus, receiving oral hypoglycemic drug therapy or insulin therapy, or having a hemoglobin A1c level ≥ 6.5%
Smoking	Current smoking or smoking cessation within 1 year
eGFR < 60 $mL\cdot min^{-1}\cdot 1.73\ m^{-2}$	eGFR <60 $mL\cdot min^{-1}\cdot 1.73\ m^{-2}$, eGFR was calculated using the modified glomerular filtration rate estimating equation for Chinese patients with chronic kidney disease
**Disease history**	
Heart failure	Previously history of heart failure
CHD	Previous history of myocardial infarction or underwent percutaneous coronary intervention or coronary artery bypass grafting
Stroke/TIA	Previously history of stroke or TIA
**Severe clinical condition at admission**	
Heart failure	Heart failure at admission

	**Definition**
Cardiogenic shock	Cardiogenic shock at admission
Cardiac arrest	Cardiac arrest at admission
**Arriving the first hospitals by ambulance**	Arriving the first hospitals by ambulance
**Medications**	
DAPT at arrival	Use of any kinds of DAPT within 24 hours of first medical contact in the PCI hospital
Aspirin	Use of Aspirin within 24 hours of first medical contact in the PCI hospital
P2Y12 inhibitors	Use of any kinds of P2Y12 inhibitors within 24 hours of first medical contact in the PCI hospital
ACEIs/ARBs at arrival	Use of any kinds of ACEIs/ARBs within 24 hours of first medical contact in the PCI hospital
β-Blockers at arrival	Use of any kinds of β-Blockers within 24 hours of first medical contact in the PCI hospital
Statins at arrival	Use of any kinds of statins within 24 hours of first medical contact in the PCI hospital
GPIIb/IIIa at arrival	Use of any kinds of GPIIb/IIIa within 24 hours of first medical contact in the PCI hospital
Anticoagulant	Non-PCI usage anticoagulant during hospitalization in the PCI hospital

Abbreviations: ACEIs/ARBs, angiotensin-converting enzyme inhibitors/angiotensin receptor blockers; CHD, coronary heart disease; DAPT, dual antiplatelet therapy; eGFR, estimated glomerular filtration rate; LDL-C, low-density lipoprotein cholesterol; GPIIb/IIIa, glycoprotein IIb/IIIa inhibitors; and TIA, transient ischemic attacks.

Supplementary Table 2. Missing rates of study variables and management of missing data (variables without any missing data are not listed in the table)

	Missing rate, % (No.)	Management of the missing data
**Age**	0.16 (150/94,623)	Imputed using sequential regression multiple imputation method implemented by IVEware software
**Sex**	0.00 (5/94,623)	
**Systolic blood pressure**	0.20 (190/94,623)	
**Diastolic blood pressure**	0.27 (255/94,623)	
**LDL-C**	11.56 (10,941/94,623)	
**Serum creatinine**	4.06 (3842/94,623)	
**Cardiac arrest at admission**	0.79 (749/94,623)	
**Cardiogenic Shock at admission**	0.70 (666/94,623)	Patients with unclear status for heart failure and cardiogenic shock were imputed by Killip class. Patients with Killip class II-III were classified as acute heart failure and patients with Killip class IV were classified as cardiogenic shock. If patients with unclear status of Killip class, we imputed the missing values using sequential regression multiple imputation method implemented by IVEware software
**Heart failure at admission**	0.73 (687/94,623)	
**Time from symptom onset to primary PCI**	17.39 (6168/35,468) *	Patients with missing value were not included for the proportion estimation
**Time from PCI hospital arrival to primary PCI**	17.46 (6191/35,468) *	Patients with missing value were not included for the proportion estimation
**Time from PCI hospital arrival to PCI for NSTEMI**	21.67 (3805/17,554) †	

Notes: * The denominator was patients with STEMI who received primary PCI. † The denominator was patients with NSTEMI who received PCI.

Abbreviations: LDL-C, low-density lipoprotein cholesterol; NSTEMI, non-ST-segment elevation myocardial infarction; and PCI, percutaneous coronary intervention.

Supplementary Table 3. The median delay in receiving early revascularization of patients with STEMI and NSTEMI transferred from another hospital and directly admitted

	**Inter-hospital transfer**	**Direct admission**	***P* value**
**STEMI**			
Symptom onset to primary PCI, hour	6.5 (4.0-11.2)	4.5 (2.7-8.5)	<0.001
Door to balloon, hour	0.8 (0.4-1.5)	1.0 (0.6-1.8)	<0.001
**NSTEMI**			
Symptom onset to timely PCI, hour	16.3 (8.7-33.0)	11.9 (5.7-25.5)	<0.001
Door to timely PCI, hour	2.8 (1.1-14.2)	3.7 (1.5-10.8)	0.002

Abbreviations: NSTEMI, non-ST-segment elevation myocardial infarction; PCI, percutaneous coronary intervention; and STEMI, ST-segment elevation myocardial infarction.

Supplementary Table 4. Characteristics of inter-hospital transfer vs. direct admission in patients with AMI in the inverse probability of treatment-weighted sample

	Inter-hospital transfer (N=28,329) *, †	Direct admission (N=41,352) *, †	SMD
**Age, year**	62.3 (12.7)	62.7 (12.8)	0.002
**Female**	10026.9 (23.8)	6743.1 (23.8)	0.001
**Medical insurance**			0.021
High reimbursement	24592.4 (58.4)	16269.4 (57.4)	
Medium reimbursement	9448.2 (22.4)	6559.1 (23.1)	
Low reimbursement	8072.0 (19.2)	5522.8 (19.5)	
**Risk factor**			
Hypertension	27294.9 (64.8)	18453.7 (65.1)	0.006
LDL-C ≥ 70mg/dL	36628.0 (87.0)	24609.5 (86.8)	0.005
Diabetes mellitus	11360.1 (27.0)	7654.3 (27.0)	0.001
Smoking	18339.4 (43.5)	12434.1 (43.9)	0.006
eGFR<60 $mL \cdot min^{-1} \cdot 1.73\ m^{-2}$	7590.8 (18.0)	5164.9 (18.2)	0.005
**Disease history**			
CHD	3945.8 (9.4)	2648.4 (9.3)	0.001
Heart failure	751.7 (1.8)	494.5 (1.7)	0.003
Stroke/TIA	3569.7 (8.5)	2387.5 (8.4)	0.002
**Severe clinical condition at admission**			
Heart failure	2792.1 (6.6)	1876.6 (6.6)	<0.001
Cardiogenic shock	1421.0 (3.4)	914.9 (3.2)	0.008
Cardiac arrest	733.6 (1.7)	503.4 (1.8)	0.003
**Arriving the first hospitals by ambulance**	3714.6 (8.8)	2398.8 (8.5)	0.013
**Medications**			
DAPT at arrival	39503.0 (93.8)	26769.8 (94.4)	0.026
Aspirin	40153.2 (95.3)	27198.3 (95.9)	0.029
P2Y12 inhibitors	40374.5 (95.9)	27230.9 (96.0)	0.009
ACEIs/ARBs at arrival	19554.1 (46.4)	13311.4 (47.0)	0.010
β-Blockers at arrival	22814.4 (54.2)	26724.4 (54.6)	0.008

	Inter-hospital transfer (N=28,329) *, †	Direct admission (N=41,352) *, †	SMD
Statins at arrival	39437.0 (93.6)	26724.4 (94.3)	0.026
GPIIb/IIIa at arrival	13967.5 (33.2)	9360.1 (33.0)	0.003
Anticoagulant	32395.9 (76.9)	21937.4 (77.4)	0.011

Notes: * Proportion, mean and medians are weighted using inverse probability of treatment weighting (IPTW).

† The IPTW sample only includes those with complete data of all covariates included in the propensity analysis.

Abbreviations: ACEIs/ARBs, angiotensin-converting enzyme inhibitors/angiotensin receptor blockers; AMI, acute myocardial infarction; CHD, coronary heart disease; DAPT, dual antiplatelet therapy; eGFR, estimated glomerular filtration rate; LDL-C, low-density lipoprotein cholesterol; GPIIb/IIIa, glycoprotein IIb/IIIa inhibitors; and TIA, transient ischemic attacks.

Supplementary Table 5. The median delay in receiving early revascularization patients with STEMI and NSTEMI who were transferred from another hospital and directly admitted, after excluding missing data

	**Inter-hospital transfer**	**Direct admission**	***P* value**
**STEMI**			
Symptom onset to primary PCI, hour	7.2 (4.8-11.7)	5.4 (3.5-9.3)	<0.001
Door to balloon, hour	1.3 (0.7-2.1)	1.5 (0.9-2.3)	<0.001
**NSTEMI**			
Symptom onset to timely PCI, hour	16.7 (8.7-34.0)	13.0 (6.4-26.5)	<0.001
Door to timely PCI, hour	2.8 (1.2-14.4)	3.5 (1.5-10.7)	0.166

Abbreviations: NSTEMI, non-ST-segment elevation myocardial infarction; PCI, percutaneous coronary intervention; and STEMI, ST-segment elevation myocardial infarction.

Supplementary Table 6. Early revascularization of patients with STEMI and NSTEMI who were transferred from another hospital and directly admitted, after excluding missing data

	Inter-hospital transfer	Direct admission	*P* value
**STEMI**			
Reperfusion	8298 (50.9)	8780 (60.6)	<0.001
Fibrinolysis	1974 (12.1)	731(5.0)	<0.001
Primary PCI	6324 (38.8)	8049 (55.5)	<0.001
DTB within 90min*	4467 (70.5)	5248 (65.2)	<0.001
**NSTEMI**			
Timely PCI†	761 (28.4)	1217 (36.2)	<0.001

Notes: * The denominator was STEMI patients who received primary PCI. † The denominator was NSTEMI patients who received PCI.

Abbreviations: DTB, door-to-balloon, NSTEMI, non-ST-segment elevation myocardial infarction; PCI, percutaneous coronary intervention; and STEMI, ST-segment elevation myocardial infarction.

Supplementary Table 7. The association between in-hospital outcomes and inter-hospital transfer vs. direct admission in patients with STEMI and NSTEMI, after excluding missing data

	Inter-hospital transfer	Direct admission	HR (95%CI) *	*P* value
**STEMI**				
MACE	2.2 (268/12,450)	2.9 (377/13,223)	0.96 (0.80-1.15)	0.674
Death	1.4 (174/12,450)	1.8 (234/13,223)	1.07 (0.87-1.33)	0.522
**NSTEMI**				
MACE	1.6 (49/2985)	2.8 (151/5463)	0.98 (0.67-1.43)	0.925
Death	1.1 (32/2985)	1.7 (91/5463)	1.07 (0.69-1.67)	0.755

Notes: * Adjusted for age, sex, medical insurance, risk factors (hypertension, LDL-C ≥ 70 mg/dL, eGFR < 60 $mL \cdot min^{-1} \cdot 1.73\ m^{-2}$, diabetes mellitus, and smoking), disease history (CHD, heart failure, and stroke/TIA), clinical condition at admission (heart failure, cardiogenic shock, cardiac arrest), time from symptom onset to PCI hospital admission, ambulance, DAPT, GPIIb/IIIa, ACEI/ARBs, β-blockers and statins at arrival, anticoagulant, year of admission, characteristics of hospital (hospital level, economic level), geographical area, fibrinolysis (only for STEMI) and PCI (primary PCI, non-primary PCI and no PCI for STEMI; timely PCI, non-timely PCI and no PCI for NSTEMI).

Abbreviations: CI, confidence interval; HR, hazard ratio; MACE, major adverse cardiovascular events; NSTEMI, non-ST-segment elevation myocardial infarction; and STEMI, ST-segment elevation myocardial infarction.

Supplementary Table 8. Investigators of CCC-ACS project

ID	Hospitals	Territories	Provinces	City	Investigator
1	Peking University First Hospital	Northern China	Beijing	Beijing	Jie Jiang
2	Beijing Anzhen Hospital, Capital Medical University	Northern China	Beijing	Beijing	Shaoping Nie, Xiaohui Liu
3	The First Affiliated Hospital of Bengbu Medical College	Eastern China	Anhui	Bengbu	Honhju Wang
4	Beijing Friendship Hospital, Capital Medical University	Northern China	Beijing	Beijing	Hongwei Li
5	The First Affiliated Hospital of Chongqing Medical University	Southwest China	Chongqing	Chongqing	Suxin Luo
6	Changhai Hospital of Shanghai	Eastern China	Shanghai	Shanghai	Xianxian Zhao
7	Xinqiao Hospital, Third Military Medical University	Southwest China	Chongqing	Chongqing	Cui Bin, Lan Huang
8	Dongguan People's Hospital	Southern China	Guangdong	Dongguan	Jianfeng Ye
9	Zhongda Hospital, Southeast University	Eastern China	Jiangsu	Nanjing	Genshan Ma
10	Gansu Provincial Hospital	Northwest China	Gansu	Lanzhou	Ping Xie
11	Guangdong General Hospital	Southern China	Guangdong	Guangzhou	Jiyan Chen
12	The First Affiliated Hospital of Guangxi Medical University	Southern China	Guangxi	Nanning	Lang Li
13	The People's Hospital of Guangxi Zhuang Autonomous Region	Southern China	Guangxi	Nanning	Yingzhong Lin
14	Panyu Hospital of Chinese Medicine	Southern China	Guangdong	Guangzhou	Jianhao Li
15	The Affiliated Hospital of Guizhou Medical University	Southwest China	Guizhou	Guiyang	Lirong Wu
16	The 2nd Affiliated Hosipital of Harbin Medical University	Northeast China	Heilongjiang	Harbin	Bo Yu
17	Navy General Hospital	Northern China	Beijing	Beijing	Tianchang Li
18	Haikou People's Hospital	Southern China	Hainan	Haikou	Moshui Chen
19	Hainan General Hospital	Southern China	Hainan	Haikou	Bin Li
20	The First Hospital of Handan	Northern China	Hebei	Handan	Shuanli Xin

ID	Hospitals	Territories	Provinces	City	Investigator
21	Hebei General Hospital	Northern China	Hebei	Shijiazhuang	Xiaoyong Qi
22	The Second Hospital of Hebei Medical University	Northern China	Hebei	Shijiazhuang	Xianghua Fu
23	The First Affiliated Hospital of Henan University of Science and Technology	Central China	Henan	Luoyang	Pingshuan Dong
24	Henan Provincial People's Hospital	Central China	Henan	Zhengzhou	Chuanyu Gao
25	Chenzhou First People's Hospital	Central China	Hunan	Chenzhou	Qiaoqing Zhong
26	Hunan Provincial People's Hospital	Central China	Hunan	Changsha	Ying Guo
27	West China Hospital of Sichuan University	Northwest China	Sichuan	Chengdu	Xiaoping Chen
28	Huai'an First People's Hospital	Eastern China	Jiangsu	Huai'an	Shuren Ma
29	The First Hospital of Jilin University	Northeast China	Jilin	Changchun	Yang Zheng
30	The Second Hospital of Jilin University	Northeast China	Jilin	Changchun	Bin Liu
31	Nanjing Drum Tower Hospital, The Affiliated Hospital of Nanjing University Medical School	Eastern China	Jiangsu	Nanjing	Biao Xu, Guangshu Han
32	Jiangsu Province Hospital	Eastern China	Jiangsu	Nanjing	Zhijian Yang
33	The 309th Hospital of Chinese People's Liberation Army	Northern China	Beijing	Beijing	Fakuan Tang, Jun Xiao
34	First Affiliated Hospital of the People's Liberation Army General Hospital	Northern China	Beijing	Beijing	Miao Tian
35	The First Affiliated Hospital of Lanzhou University	Northwest China	Gansu	Lanzhou	Zheng Zhang
36	The First Affiliated Hospital of Liaoning Medical University	Northeast China	Liaoning	Jinzhou	Guizhou Tao
37	China Meitan General Hospital	Northern China	Beijing	Beijing	Di Wu
38	The First Affiliated Hospital to Nanchang University	Eastern China	Jiangxi	Nanchang	Zeqi Zheng
39	The Second Affiliated Hospital to Nanchang University	Eastern China	Jiangxi	Nanchang	Xiaoshu Cheng
40	Nanfang Hospital of Southern Medical University	Southern China	Guangdong	Guangzhou	Yuqing Hou

ID	Hospitals	Territories	Provinces	City	Investigator
41	Inner Mongolia People's Hospital	Northern China	Inner Mongolia	Hohhot	Xingsheng Zhao
42	Affiliated Hospital of Ningxia Medical University	Northwest China	Ningxia	Yinchuan	Shaobin Jia
43	People’s Hospital of Qinghai Province	Northwest China	Qinghai	Xining	Rong Chang
44	Binzou City Center Hospital	Eastern China	Shandong	Binzhou	Lijun Meng
45	Shanxi Provincial People's Hospital	Northern China	Shanxi	Taiyuan	Chunlin Lai
46	Shanxi Cardiovascular Hospital	Northern China	Shanxi	Taiyuan	Bao Li
47	The Second Hospital of Shanxi Medical University	Northern China	Shanxi	Taiyuan	Zhiming Yang
48	The Ninth Hospital Affiliated to Shanghai Jiaotong University School of Medicine	Eastern China	Shanghai	Shanghai	Changqian Wang
49	Shanghai Sixth People's Hospital	Eastern China	Shanghai	Shanghai	Shixin Ma
50	Tongren Hospital Affiliated to Shanghai Jiaotong University School of Medicine	Eastern China	Shanghai	Shanghai	Li Jiang
51	The General Hospital of Shenyang Military Region	Northeast China	Liaoning	Shenyang	Yaling Han
52	The Third Hospital of Shijiazhuang	Northern China	Hebei	Shijiazhuang	Zhenguo Ji
53	North Jiangsu People's Hospital	Eastern China	Jiangsu	Yangzhou	Shenghu He
54	General Hospital of TISCO	Northern China	Shanxi	Taiyuan	Huifeng Wang
55	Tianjin Chest Hospital	Northern China	Tianjin	Tianjin	Yin Liu
56	Teda International Cardiovascular Hospital	Northern China	Tianjin	Tianjin	Wenhua Lin
57	Tianjin Medical University General Hospital	Northern China	Tianjin	Tianjin	Yuemin Sun
58	Wuxi People's Hospital	Eastern China	Jiangsu	Wuxi	Zhenyu Yang
59	The First Affiliated Hospital of Xi’an Jiaotong University	Northwest China	Shaanxi	Xi'an	Zuyi Yuan
60	Xijing Hospital	Northwest China	Shaanxi	Xi'an	Ling Tao

ID	Hospitals	Territories	Provinces	City	Investigator
61	Southwest Hospital, Third Military Medical University	Southwest China	Chongqing	Chongqing	Zhiyuan Song
62	Hospital of Xinjiang Production & Construction Corps	Northwest China	Xinjiang	Urumchi	Junming Liu
63	The First Teaching Hospital of Xinjiang Medical University	Northwest China	Xinjiang	Urumchi	Yitong Ma
64	Xinjiang Uygur Autonomous Region People's Hospital	Northwest China	Xinjiang	Urumchi	Guoqing Li
65	The Affiliated Hospital of Xuzhou Medical College	Eastern China	Jiangsu	Xuzhou	Zhirong Wang
66	People's Hospital of Yuxi City	Southwest China	Yunnan	Yuxi	Yinglu Hao
67	The Second People's Hospital of Yunnan Province	Southwest China	Yunnan	Kunming	Minghua Han
68	Sir Run Run Shaw Hospital, College of Medicine, Zhejiang University	Eastern China	Zhejiang	Hangzhou	Guosheng Fu
69	The Second Affiliated Hospital of Zhengzhou University	Central China	Henan	Zhengzhou	Yulan Zhao
70	The First Affiliated Hospital of Zhengzhou University	Central China	Henan	Zhengzhou	Ling Li
71	The Third Xiangya Hospital of Central South University	Central China	Hunan	Changsha	Weihong Jiang
72	Sun Yat-sen Memorial Hospital, Sun Yat-sen University	Southern China	Guangdong	Guangzhou	Jingfeng Wang
73	The Military General Hospital of Beijing PLA	Northern China	Beijing	Beijing	Junxia Li
74	Baogang Hospital	Northern China	Inner Mongolia	Baotou	Yongdong Li
75	Zhejiang Provincial Hospital of TCM	Eastern China	Zhejiang	Hangzhou	Wei Mao
76	Affiliated Hospital of Qinghai University	Northwest China	Qinghai	Xining	Weijun Liu
77	Anhui Provincial Hospital	Eastern China	Anhui	Hefei	Likun Ma
78	Anyang District Hospital	Central China	Henan	Anyang	Hui Liu
79	The Third the People's Hospital of Bengbu	Eastern China	Anhui	Bengbu	Gengsheng Sang
80	Cangzhou Central Hospital	Northern China	Hebei	Cangzhou	Zesheng Xu

ID	Hospitals	Territories	Provinces	City	Investigator
81	The First People's Hospital of Changde	Central China	Hunan	Changde	Yi Huang
82	Dalian Municipal Central Hospital	Northeast China	Liaoning	Dalian	Hailong Lin
83	The Second hospital of Dalian Medical University	Northeast China	Liaoning	Dalian	Peng Qu
84	The First Affiliated hospital of Dalian Medical University	Northeast China	Liaoning	Dalian	Yanzong Yang
85	Fujian Provincial Hospital	Eastern China	Fujian	Fuzhou	Yansong Guo
86	Longyan First Hospital	Eastern China	Fujian	Longyan	Kaihong Chen
87	The First Affiliated Hospital of Fujian Medical University	Eastern China	Fujian	Fuzhou	Jinzi Su
88	Affiliated Hospital of Guangdong Medical College	Southern China	Guangdong	Guangzhou	Keng Wu
89	Guangzhou Red Cross Hospital	Southern China	Guangdong	Guangzhou	Tongguo Wu
90	The First Affiliated Hospital of Guangzhou Medical College	Southern China	Guangdong	Guangzhou	Wei Wang
91	The Third Affiliated Hospital of Guangzhou Medical College	Southern China	Guangdong	Guangzhou	Ximing Chen
92	Guizhou Provincial People's Hospital	Northwest China	Guizhou	Guiyang	Qiang Wu
93	The Central Hospital of Zhoukou	Central China	Henan	Zhoukou	Hualing Liu
94	The Central Hospital of Jilin	Northeast China	Jilin	Changchun	Shuangbin Li
95	The First People's Hospital of Jining	Eastern China	Shandong	Jining	Xiaofei Sun
96	Affiliated Hospital of Jiangsu University	Eastern China	Jiangsu	Zhenjiang	Jinchuan Yan
97	Jiangxi Provincial People's Hospital	Eastern China	Jiangxi	Nanchang	Lang Ji
98	The People's Hospital of Liaoning Province	Northeast China	Liaoning	Shenyang	Zhanquan Li
99	The First Affiliated Hospital of Liaoning University of Traditional Chinese Medicine	Northeast China	Liaoning	Shenyang	Ping Hou
100	Liaocheng People's Hospital	Eastern China	Shandong	Liaocheng	Chunyan Zhang

ID	Hospitals	Territories	Provinces	City	Investigator
101	Linyi People's Hospital	Eastern China	Shandong	Linyi	Zhihong Ou
102	Mudanjiang Cardiovascular Disease Hospital	Northeast China	Heilongjiang	Mudanjiang	Jianwen Liu
103	The First People's Hospital of Nanning City	Southern China	Guangxi	Nanning	Jinru Wei
104	Ningxia People's Hospital	Northwest China	Ningxia	Yinchuan	Hong Luan
105	Qingdao Municipal Hospital	Eastern China	Shandong	Qingdao	Jun Guan
106	Quanzhou First Hospital	Eastern China	Fujian	Quanzhou	Rong Lin
107	The First Affiliated Hospital of Xiamen University	Eastern China	Fujian	Xiamen	Qiang Xie
108	Xiamen Cardiovascular Disease Hospital	Eastern China	Fujian	Xiamen	Yan Wang
109	Qilu Hospital of Shandong University	Eastern China	Shandong	Jinan	Jifu Li
110	Yantaishan hospital	Eastern China	Shandong	Yantai	Juexin Fan
111	Zhongshan Hospital Affiliated to Fudan University	Eastern China	Shanghai	Shanghai	Junbo Ge
112	Shanghai East Hospital Affiliated to Tongji University	Eastern China	Shanghai	Shanghai	Xuebo Liu
113	The Central Hospital of Shaoyang	Central China	Hunan	Shaoyang	Zewei Ouyang
114	Central Hospital Affiliated to Shenyang Medical College	Northeast China	Liaoning	Shenyang	Man Zhang, Kaiming Chen
115	The First Affiliated Hospital of Soochow University	Eastern China	Jiangsu	Suzhou	Xiangjun Yang
116	The Second Affiliated Hospital of Soochow University	Eastern China	Jiangsu	Suzhou	Weiting Xu
117	The Central Hospital of Taiyuan	Northern China	Shanxi	Taiyuan	Xiaoping Chen
118	Tangshan Gongren Hospital	Northern China	Hebei	Tangshan	Zheng Ji
119	The First Affiliated Hospital of Wannan Medical College	Eastern China	Anhui	Wuhu	Xingsheng Tang
120	The First Affiliated Hospital of Wenzhou Medical University	Eastern China	Zhejiang	Wenzhou	Weijian Huang
121	Wuzhou People's Hospital	Southern China	Guangxi	Wuzhou	Shaowu Ye

ID	Hospitals	Territories	Provinces	City	Investigator
122	Renmin Hospital of Wuhan University	Central China	Hubei	Wuhan	Hong Jiang
123	Xiangtan City Central Hospital	Central China	Hunan	Xiangtan	Lilong Tang
124	The Central Hospital of Xuzhou	Eastern China	Jiangsu	Xuzhou	Peiying Zhang
125	Affiliated Hospital of Yan'an University	Northwest China	Shaanxi	Yan'an	Xiaochuan Ma
126	Yancheng Third People's Hospital	Eastern China	Jiangsu	Yancheng	Chunyang Wu
127	Yangzhou First People's Hospital	Eastern China	Jiangsu	Yangzhou	Aihua Li
128	Yichang Central Hospital	Central China	Hubei	Yichang	Jiawang Ding
129	The First People's Hospital of Yunnan Province (Kunhua Hospital)	Northwest China	Yunnan	Kunming	Hong Zhang
130	Hospital 463 of Chinese People's Liberation Army	Northeast China	Liaoning	Shenyang	Bosong Yang
131	The First Affiliated Hospital of China Medical University	Northeast China	Liaoning	Shenyang	Yingxian Sun
132	The Fourth Affiliated Hospital of China Medical University	Northeast China	Liaoning	Shenyang	Yuanzhe Jin
133	The Second Xiangya Hospital of Central South University	Central China	Hunan	Changsha	Daoquan Peng
134	Xiangya Hospital Central South University	Central China	Hunan	Changsha	Tianlun Yang
135	Zhoushan People's Hospital	Eastern China	Zhejiang	Zhoushan	Guoxiong Chen
136	Chengdu Third People's Hospital	Northwest China	Sichuan	Chengdu	Jiong Tang
137	Tangdu Hospital of The Fourth Military Medical University	Northwest China	Shaanxi	Xi'an	Xue Li
138	The First Hospital of Haerbin City	Northeast China	Heilongjiang	Harbin	Lin Wei
139	The First Affiliated Hospital of Jiamusi University	Northeast China	Heilongjiang	Jiamusi	Zhaofa He
140	The Central Hospital of Panzhihua	Northwest China	Sichuan	Panzhihua	Dawen Xu
141	The First Hospital of Qiqihaer City	Northeast China	Heilongjiang	Qiqihaer	Gang Xu

ID	Hospitals	Territories	Provinces	City	Investigator
142	Wuhan Asia Heart Hospital	Central China	Hubei	Wuhan	Xi Su
143	Sichuan Provincial People's Hospital	Northwest China	Sichuan	Chengdu	Jianhong Tao
144	The Central Hospital of Mianyang	Northwest China	Sichuan	Mianyang	Caidong Luo
145	The First Hospital of Jiamusi	Northeast China	Heilongjiang	Jiamusi	Guixia Zhang
146	Huaibei Miners General Hospital	Eastern China	Anhui	Huaibei	Zhenqi Su
147	Beijing Tsinghua Changgung Hospital	Northern China	Beijing	Beijing	Ping Zhang
148	Chongqing Hechuan District People's Hospital	Southwest China	Chongqing	Chongqing	Xin Tang
149	Yuzhou City Central Hospital	Central China	Henan	Xuchang	Qinfeng Su
150	Jianshui County People's Hospital	Southwest China	Yunnan	Honghe	Weiqing Fan
151	Dunhua City Hospital	Northeast China	Jilin	Yanbian	Fanju Meng
152	Shenyang City Electricity Central Hospital	Northeast China	Liaoning	Shenyang	Jing Xu
153	Shanghai Jingan District Shibei Hospital	Eastern China	Shanghai	Shanghai	Bin Wang
154	Beijing Fangshan District First Hospital	Northern China	Beijing	Beijing	Xuemei Peng
155	Hebei Daming County People's Hospital	Northern China	Hebei	Handan	Haiping Guo
156	Jiangsu Binhai County People's Hospital	Eastern China	jiangsu	Yancheng	Yonglin Zhang
157	The First People's Hospital of Longquanyi District	Southwest China	Sichuan	Chengdu	Wei Tuo
158	Guangxi Hengxian County People's Hospital	Southern China	Guangxi	Nanning	Xianan Zhang
159	Hunan Changsha County First People's Hospital	Central China	Hunan	Changsha	Siding Wang
160	People's Hospital of Wugang	Central China	Hunan	Shaoyang	JiaoMei Yang
161	Longhui County People's Hospital	Central China	Hunan	Shaoyang	Xiaojun Wang
162	Heilongjiang Fujin City Central Hospital	Northeast China	Heilongjiang	Jiamusi	Jiyan Yin

ID	Hospitals	Territories	Provinces	City	Investigator
163	Dalian Fourth People’s Hospital	Northeast China	Liaoning	Dalian	Huifang Zhang
164	General Hospital of Guangzhou Military Command	Southern China	Guangdong	Guangzhou	Yanlie Zheng
165	The First People’s Hospital of Horqin District, Tongliao City	Northern China	Inner Mongolia	Tongliao	Junping Fang
166	Guiyang Sixth People’s Hospital	Southwest China	Guizhou	Guiyang	Kalan Luo
167	Geological Mining Hospital of Hunan Province	Central China	Hunan	Changsha	Naiyi Liang
168	Zhangzhou Municipal Hospital of Fujian Province	Eastern China	Fujian	Zhangzhou	Changyong Liu
169	Jining City Yanzhou District People’s Hospital	Eastern China	Shandong	Jining	Jian Yang
170	The People’s Hospital Feixian	Eastern China	Shandong	Linyi	Honghua Deng
171	Tangshan City Fengrun District People’s Hospital	Northern China	Hebei	Tangshan	Lin Wang
172	Qian’an People’s Hospital	Northern China	Hebei	Tangshan	Yuheng Yang
173	Yuzhong County People’s Hospital	Northwest China	Gansu	Lanzhou	Xiaowei Peng
174	Baiyin Cite Center Hospital	Northwest China	Gansu	Baiyin	Fang Zhao
175	Mingguang People’s Hospital	Eastern China	Anhui	Chuzhou	Yong Li
176	Xihua County People’s Hospital	Central China	Henan	Zhoukou	Chuntong Wang
177	Zhalantun People’s Hospital	Northern China	Inner Mongolia	Hulunbeier	Yuhua Zhu
178	Fengrun District Second People’s Hospital	Northern China	Hebei	Tangshan	Jingshan Zhao
179	Zhangping City Hospital	Eastern China	Fujian	Longyan	Jinxing Yi
180	The Eight Affiliated Hospital, Sun Yat-sen University	Southern China	Guangdong	Guangzhou	Nan Jia
181	The Second Affiliated Hospital of Qiqihar Medical Hospital	Northeast China	Heilongjiang	Qiqihar	Yanli Wang
182	Fuqing Cite Hospital	Eastern China	Fujian	Fuqing	Ping Chen
183	Wuhan University of Science and Technology Hospital	Central China	Hubei	Wuhan	Jing Hu

ID	Hospitals	Territories	Provinces	City	Investigator
184	Baotou City Center Hospital	Northern China	Inner Mongolia	Baotou	Ruiping Zhao
185	Shanghai Jiading District Center Hospital	Eastern China	Shanghai	Shanghai	Xia Chen
186	Datong City Second People’s Hospital	Northern China	Shanxi	Datong	Xiaoqin Zhang
187	Binyang People’s Hospital	Southern China	Guangxi	Nanning	Fudong Gan
188	Deqing People’s Hospital	Eastern China	Zhejiang	Huzhou	Fangfang Huang
189	Xinmi people’s hospital	Central China	Henan	Zhengzhou	Xiaolei Li
190	Dongguan Changping hospital	Southern China	Guangdong	Dongguan	Haiyun Lin
191	Gongyi people’s hospital	Central China	Henan	Zhengzhou	Tianmin Du
192	Ye County people’s hospital	Central China	Henan	Pingdingshan	Jie Yang
193	The second people’s hospital of Mengcheng	Eastern China	Anhui	Bozhou	Pengfei Zhang
194	Nanpi People’s Hospital	Northern China	Hebei	Cangzhou	Hui Dong
195	Shimen People’s Hospital	Central China	Hunan	Changde	Chuanliang Liang
196	Tieli People’s Hospital	Northeast China	Heilongjiang	Yichun	Yanbo Niu
197	Sihui People’s Hospital	Southern China	Guangdong	Zhaoqing	Yuehua Huang
198	Chest Hospital of Xinjiang Uygur Autonomous Region	Northwest China	Xinjiang	Urumchi	Dongsheng Chai
199	Beian First People’s Hospital	Northeast China	Heilongjiang	Heihe	Dongyan Li
200	Zunhua People’s Hospital	Northern China	Hebei	Tangshan	Xiaoli Yang
201	Lujiang People’s Hospital	Eastern China	Anhui	Hefei	Qichun Wang
202	Qinyang People’s Hospital	Central China	Henan	Jiaozuo	Xiaowen Ma
203	Longmen People’s Hospital	Southern China	Guangdong	Huizhou	Yingchao Luo
204	Quyang Renji Hospital	Northern China	Hebei	Baoding	Congliang Zhang

ID	Hospitals	Territories	Provinces	City	Investigator
205	Nenjiang People's Hospital	Northeast China	Heilongjiang	Heihe	Shuhua Zhang
206	Longjiang First People's Hospital	Northeast China	Heilongjiang	Qiqihar	Yuhuan Shi
207	Li County Hospital of Traditional Chinese Medicine	Central China	Hunan	Changde	Songbai Li
208	Luan County People's Hospital	Northern China	Hebei	Tangshan	Guo Li
209	Yulong Hospital	Southwest China	Yunnan	Lijiang	Zeyuan He
210	Huining People's Hospital	Northwest China	Gansu	Baiyin	Jiabin Xi
211	Yuncheng Hospital	Eastern China	Shandong	Heze	Jinglan Diao
212	Hepu People's Hospital	Southern China	Guangxi	Beihai	Meisheng Lai
213	Duzishan Petrochemical Hospital	Northwest China	Xinjiang	Karamay	Shuqiu Qu
214	Guiding People's Hospital	Southwest China	Guizhou	Qinan	Guoduo Chen
215	People's Hospital of Rongchang District	Southwest China	Chongqing	Chongqing	Jie Chen
216	Ningbo First Hospital	Eastern China	Zhejiang	Ningbo	Huimin Chu
217	Ledong Second People's Hospital	Southern China	Hainan	Ledong	Xiufeng Chen
218	Guang'an People's Hospital	Southwest China	Sichuan	Guang'an	Tian Tuo
219	Linfen People's Hospital	Northern China	Shanxi	Linfen	Junping Deng
220	People's Hospital of Bozhou District	Southwest China	Guizhou	Zunyi	Shengyong Chen
221	Dianjiang People's Hospital	Southwest China	Chongqing	Chongqing	Yang Yu
222	First Affiliated Hospital of Harbin Medical University.	Northeast China	Heilongjiang	Harbin	Yue Li
223	Yiniang Hospital	Southwest China	Yunnan	Kunming	Liqiong Yang
224	Haidong Ping'an District Hospital of Traditional Chinese Medicine	Northwest China	Qinghai	Haidong	Guoqin Xin

ID	Hospitals	Territories	Provinces	City	Investigator
225	Ningjin People's Hospital	Eastern China	Shandong	Dezhou	Tao Zhang
226	Yutian Hospital	Northern China	Hebei	Tangshan	Xiaoyun Feng
227	Yanting People's Hospital	Southwest China	Sichuan	Mianyang	Mingcheng Bai
228	The Fourth Affiliated Hospital Zhejiang University School of Medicine	Eastern China	Zhejiang	Yiwu	Shudong Xia
229	Zhongda Hospital, Southeast University (Jiangbei)	Eastern China	Jiangsu	Nanjing	Jiayi Tong
230	Wuxi Xishan People's Hospital	Eastern China	Jiangsu	Wuxi	Xudong Li
231	Dongfeng Hospital	Northeast China	Jilin	Liaoyuan	Wei Liu
232	Zhijin People's Hospital	Southwest China	Guizhou	Bijie	Zhongshan Wang
233	Huaiyang People's Hospital	Central China	Henan	Zhoukou	Li Wei
234	Suizhou Central Hospital	Central China	Hubei	Suizhou	Fengwei Li
235	Tonglu First People's Hospital	Eastern China	Zhejiang	Hangzhou	Xiaolan Li
236	Xiantao First People's Hospital	Central China	Hubei	Xiantao	Dongmei Zhu
237	Honghu People's Hospital	Central China	Hubei	Jingzhou	Hong Liu
238	Affilioted Hospital of North Sichuan Medical College	Northwest China	Sichuan	Nanchong	Zhan Lv
239	Guangyuan Central Hospital	Northwest China	Sichuan	Guangyuan	Bing Fu
240	Dazhou Central Hospital	Northwest China	Sichuan	Dazhou	Yong Guo
241	Nanchong Central Hospital	Northwest China	Sichuan	Nanchong	Tao Liu

Supplementary Figure 1. The map of regional economic level of China

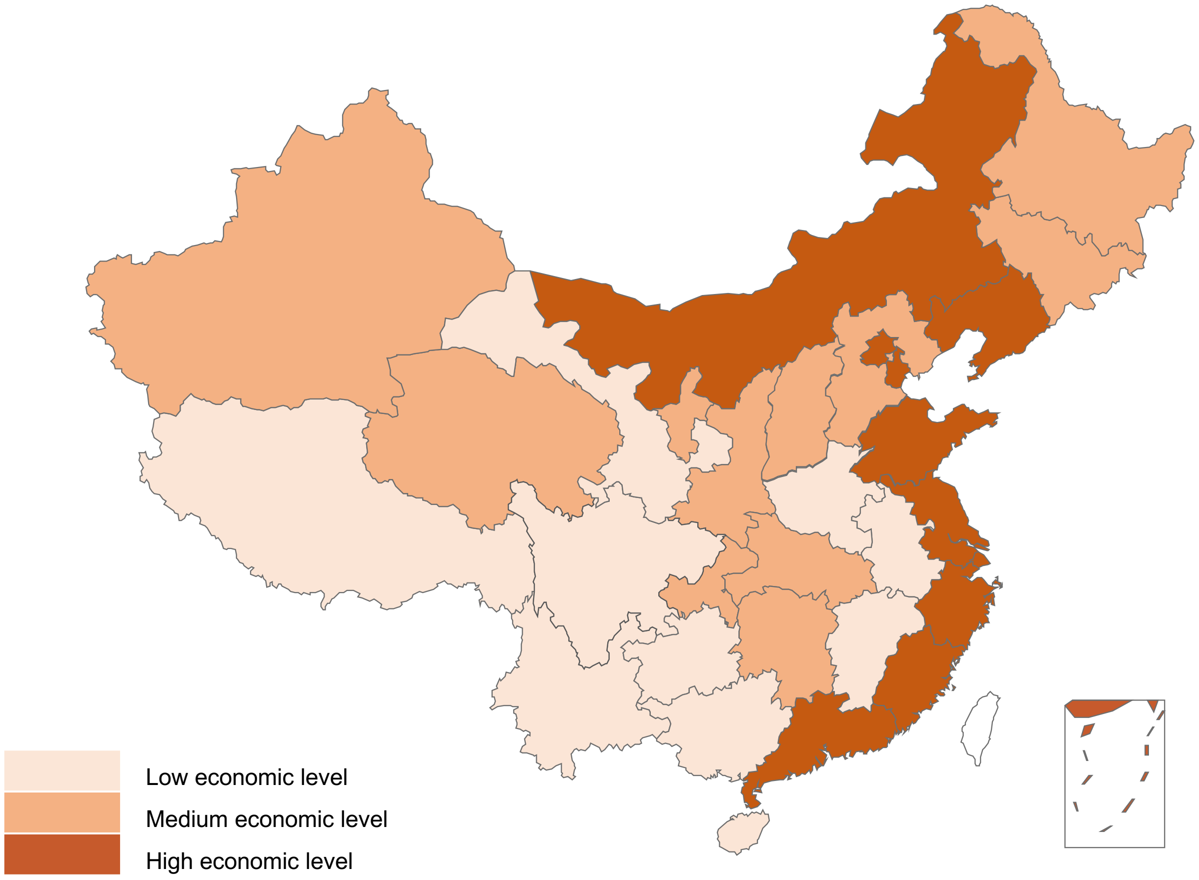

Supplementary Figure 2. Proportion of patients with STEMI and NSTEMI in hospitals with different inter-hospital transfer rates

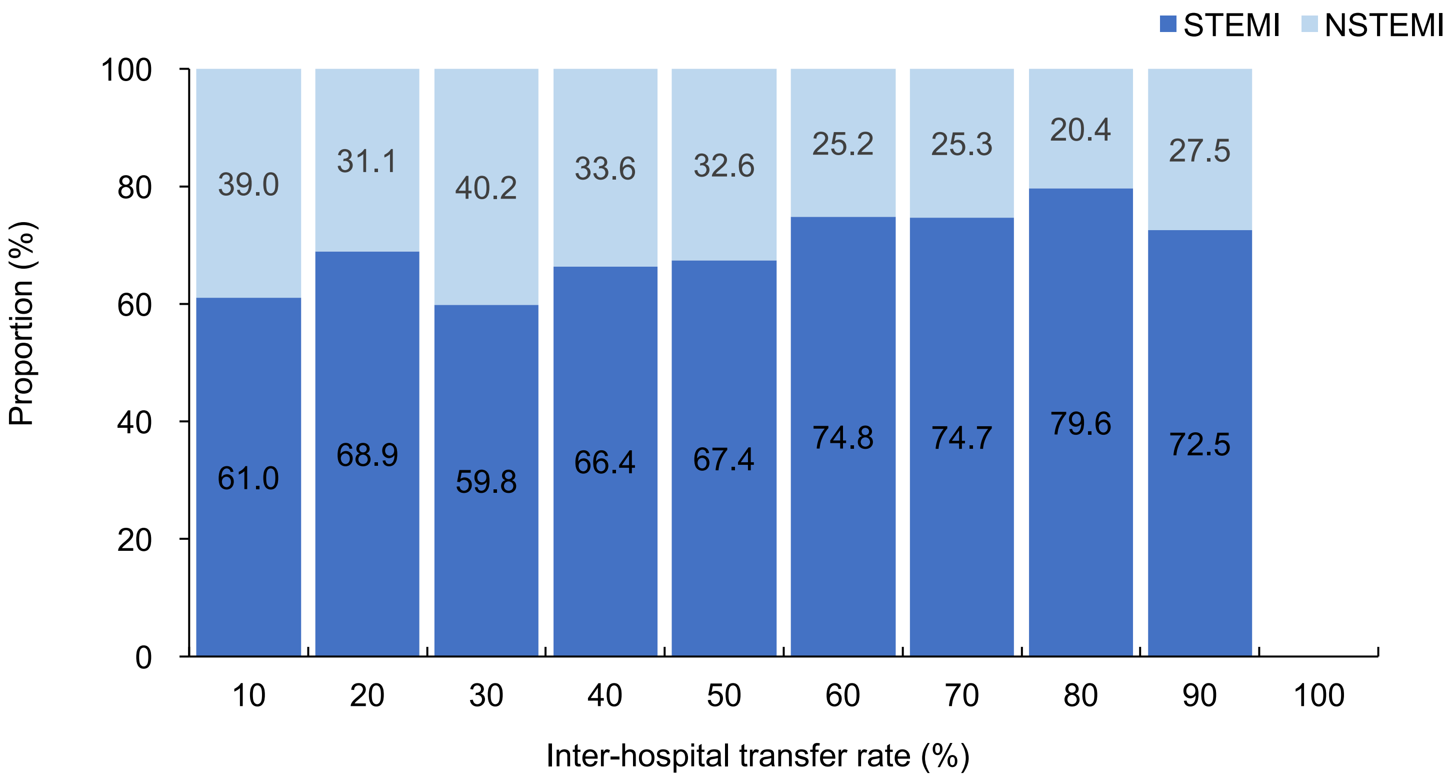

Abbreviations: NSTEMI, non-ST-segment elevation myocardial infarction; and STEMI, ST-segment elevation myocardial infarction.
